# Intratracheal Administration of Chloroquine-Loaded Niosomes Minimize Systemic Drug Exposure

**DOI:** 10.3390/pharmaceutics13101677

**Published:** 2021-10-14

**Authors:** Hesham A. Saafan, Kamilia M. Ibrahim, Yasmeena Thabet, Sara M. Elbeltagy, Rana A. Eissa, Ashraf H. Ghaleb, Fathy Ibrahim, Mahmoud Elsabahy, Noura G. Eissa

**Affiliations:** 1 School of Biotechnology, Badr University in Cairo, Cairo 11829, Egypt; heshamalaasaafan647@gmail.com (H.A.S.); Kamiliamagdy@gmail.com (K.M.I.); yasmeena.thabet@med.fsu.edu (Y.T.); sara.mohamedelbeltagy@gmail.com (S.M.E.); Rana.Ahmed@buc.edu.eg (R.A.E.); 2 Department of Biomedical Sciences, Florida State University College of Medicine, Tallahassee, FL 32304, USA; 3 Galala University, Galala, Suez 43527, Egypt; ashraf.ghaleb@gu.edu.eg; 4 Department of Surgery, Faculty of Medicine, Cairo University, Cairo 12613, Egypt; 5 International Center for Bioavailability, Pharmaceutical and Clinical Research, Obour City 11828, Egypt; fibrahim@icbr.info; 6 Department of Pharmaceutics and Industrial Pharmacy, Faculty of Pharmacy, Al-Azhar University, Cairo 11651, Egypt; 7 Department of Pharmaceutics and Industrial Pharmacy, Faculty of Pharmacy, Zagazig University, Zagazig 44519, Egypt; nouraeissa@zu.edu.eg

**Keywords:** chloroquine, niosomes, inhalational, intratracheal, micromeritic, angle of repose, flowing properties

## Abstract

Pulmonary administration provides a useful alternative to oral and invasive routes of administration while enhancing and prolonging the accumulation of drugs into the lungs and reducing systemic drug exposure. In this study, chloroquine, as a model drug, was loaded into niosomes for potential pulmonary administration either via dry powder inhalation or intratracheally. Chloroquine-loaded niosomes have been prepared and extensively characterized. Furthermore, drug-loaded niosomes were lyophilized and their flowing properties were evaluated by measuring the angle of repose, Carr’s index, and Hausner ratio. The developed niosomes demonstrated a nanosized (100–150 nm) spherical morphology and chloroquine entrapment efficiency of ca. 24.5%. The FT-IR results indicated the incorporation of chloroquine into the niosomes, whereas in vitro release studies demonstrated an extended-release profile of the drug-loaded niosomes compared to the free drug. Lyophilized niosomes exhibited poor flowability that was not sufficiently improved after the addition of lactose or when cryoprotectants were exploited throughout the lyophilization process. In vivo, intratracheal administration of chloroquine-loaded niosomes in rats resulted in a drug concentration in the blood that was 10-fold lower than the oral administration of the free drug. Biomarkers of kidney and liver functions (i.e., creatinine, urea, AST, and ALT) following pulmonary administration of the drug-loaded nanoparticles were of similar levels to those of the control untreated animals. Hence, the use of a dry powder inhaler for administration of lyophilized niosomes is not recommended, whereas intratracheal administration might provide a promising strategy for pulmonary administration of niosomal dispersions while minimizing systemic drug exposure and adverse reactions.

## 1. Introduction

Pulmonary drug delivery provides a useful alternative to oral and invasive routes of administration for systemic and local effects, particularly for drugs utilized for the management of respiratory diseases. The large alveolar surface area and low thickness of the epithelial barrier, as well as bypassing the hepatic first-pass effect, allow for reduced frequency of administration, minimal side effects, and enhanced drug bioavailability [1,2,3]. Moreover, employing nanomaterials in pulmonary drug delivery allows uniform drug distribution in the lungs [2,4,5]. Our group has previously demonstrated the retention of a near-infrared probe loaded into biodegradable polyphosphoester shell crosslinked knedel-like nanoparticles following intratracheal administration in mice, as demonstrated by the lung extravasation half-lives of 4 h and 8 d for the free dye and the dye loaded into the nanoparticles, respectively [6].

Niosomes are nanoscale vesicular drug delivery systems that have been widely explored for the delivery of various therapeutics via different routes of administration, as previously demonstrated by our group [7,8] and others [9,10,11]. The vesicles are mainly comprised of non-ionic amphiphilic surfactants, usually mixed with cholesterol to allow for enhanced rigidity and stability of the system [12,13].

Chloroquine, an antimalarial drug, has been selected as a model drug for encapsulation into the developed niosomes. Chloroquine has been proposed as a potential therapeutic for coronavirus disease 2019 (COVID-19), due to its ability to inhibit severe acute respiratory syndrome coronavirus 2 (SARS-Cov-2) viral entry, transport, and post-entry events [14,15,16,17]. Recently, the COVID-19 Treatment Guidelines Panel recommended against the use of chloroquine for the treatment of COVID-19 in hospitalized patients and non-hospitalized patients, following oral administration of the drug [18]. Localized (i.e., pulmonary) administration of drugs might provide an opportunity to improve efficacy and to reduce systemic side effects by decreasing plasma concentration compared to oral administration. No previous studies have examined the ability of nanomaterials to reduce systemic exposure of chloroquine following pulmonary administration.

The current study aims to develop chloroquine-loaded niosomes for potential pulmonary administration either via dry powder inhalation or intratracheally. The purpose of the study was to examine the ability of the chloroquine-loaded niosomes to reduce blood concentration following pulmonary administration as compared to the oral administration of the free drug. The loaded niosomes have been extensively characterized by dynamic light scattering, transmission electron microscopy, Fourier transform-infrared spectroscopy, and thermogravimetric analysis. Drug entrapment efficiency and in vitro release kinetics have been also studied. Micromeritic properties (angle of repose, Carr’s index, and Hausner ratio) of the lyophilized drug-loaded niosomes have been evaluated to assess the feasibility of their in vivo administration by utilizing a dry powder inhaler. In vivo evaluation of the chloroquine-loaded niosomes has been tested in rats, and preliminary systemic drug exposure and biocompatibility have been assessed.

## 2. Materials and Methods

### 2.1. Materials

Chloroquine phosphate (purity > 99%) was obtained from the Medical Union Pharmaceuticals (Ismailia, Egypt). Polyoxyethylene sorbitan monopalmitate (Tween^®^ 40), sorbitan monostearate (Span^®^ 60), and cholesterol were purchased from Sigma-Aldrich (St. Louis, MO, USA). Chloroform (HPLC grade) was purchased from El-Nasr Pharmaceutical Co. (Cairo, Egypt). Mannitol was kindly supplied from EIPICO (10th of Ramadan City, Egypt). Trehalose was purchased from Loba Chemie (Mumbai, India). d-(+)-lactose monohydrate was purchased from MP Biomedicals, LLC (Illkirch-Graffenstaden City, France). Disodium hydrogen phosphate and potassium dihydrogen phosphate were purchased from Adwic, El-Nasr Chemical Co. (Cairo, Egypt). Dialysis tubing membrane, molecular weight cut-off 12,000–14,000 Da, was purchased from Carolina Biological Supply Co. (Burlington, NC, USA). All chemicals were used without any further purification.

### 2.2. Preparation of Niosomes

Drug-loaded niosomes were prepared using the thin-film hydration technique, as previously described [7]. Briefly, cholesterol, Span^®^ 60, and Tween^®^ 40 (molar ratio of 2:1:1, respectively) were dissolved in 3 mL chloroform. The organic solvent was then evaporated under reduced pressure using a rotary evaporator (Buch 200, BÜCHI Labortechnik AG, Flawil, Switzerland) at 40 °C at 110 rpm for 30 min. The formed dried thin lipid film was then hydrated with 10 mL of chloroquine dissolved in phosphate-buffered saline (PBS) (1 mg/mL) at pH 6.9 for 60 min at 200 rpm to allow for the formation of the chloroquine-loaded niosomes. To prepare empty niosomes, the same procedures were utilized, except for using 10 mL of PBS for hydration instead of the chloroquine drug solution. The samples were sonicated by a probe sonicator (Branson Digital Sonifier SFX 250, St. Louis, MO, USA) for 1 min (50 Hz, while turning the sonicator off and on every 2 s) to obtain niosomes of homogenous size distribution. Then, samples were centrifuged at 13,400 rpm for 30 min to allow the formation of niosomal pellets. The pellets were then washed with PBS, and the purified pellets were diluted with PBS and vortexed to form the niosomal dispersion.

### 2.3. Characterizations of Niosomes

#### 2.3.1. Dynamic Light Scattering

Mean particle size, particle size distribution (polydispersity index, PDI), and zeta potential of empty and chloroquine-loaded niosomes were evaluated using dynamic light scattering technique (Zetasizer Nano ZS, Malvern Instruments, Worcestershire, UK). An amount of 1 mL of freshly prepared samples (empty and loaded) was diluted to 10 mL with PBS and sonicated using a water-bath sonicator for 5 min before measurements.

#### 2.3.2. Transmission Electron Microscopy

Transmission electron microscopy (JEM-2100 Plus, Electron Microscope, JEOL, Tokyo, Japan) was used to study the morphology of the dispersed particles of empty and drug-loaded niosomes. One drop of the niosomal formulation was diluted 10 times and deposited onto carbon-coated copper grids and the excess liquid was removed by a filter paper. The vesicles were then stained with 2% phosphotungstic acid (*w*/*v*) and allowed to dry prior to imaging. Transmission electron microscopy (TEM) imaging was conducted at an accelerating voltage of 80 kV and the images were analyzed via ImageJ (NIH, v. 1.50i).

#### 2.3.3. Fourier Transform-Infrared Spectroscopy

The Fourier transform-infrared spectrophotometer (FTIR-8400_S_, Shimadzu, Japan) was used to record the characteristic peaks of the selected samples (chloroquine, chloroquine-loaded niosomes, and empty niosomes). Nearly 10 mg powder of each sample was mixed with potassium bromide (spectroscopic grade) and compressed into discs using hydraulic press before scanning from 4000 to 400 cm^−1^.

#### 2.3.4. Thermogravimetric Analysis

The thermal behaviors of cholesterol, chloroquine, Span^®^ 60, empty niosomes, and chloroquine-loaded niosomes were studied using thermogravimetric analysis (TGA). This test was carried out using simultaneous DTA–TGA thermal analyzer apparatus (DTA-60H, Shimadzu Corporation, Tokyo, Japan). The samples (ca. 2 mg) were sealed in aluminium pans and heated over a temperature range of 30–300 °C at a constant rate of 10 °C/min under nitrogen purge (ca. 40 mL/min).

### 2.4. Evaluation of the Entrapment Efficiency

To determine the encapsulation efficiency of the chloroquine-loaded niosomes, a freshly prepared drug-loaded niosomal dispersion was centrifuged at 13,400 rpm for 30 min at room temperature. The concentration of unentrapped chloroquine in the supernatant was detected spectrophotometrically at *λ*_max_ 343 nm (Peak Instruments Inc., Houston, TX, USA), and the percentage of entrapment efficiency was calculated according to Equation (1) [9,10]:

(1) $$\begin{array}{l} Entrapment\;Efficiency\;\left(\%\right)\\ =\frac{Total\;amount\;of\;initially\;added\;Chloroquine\;-\;Unentrapped\;Chloroquine\;}{Total\;amount\;of\;initially\;added\;Chloroquine}\\ \times 100 \end{array}$$

### 2.5. In Vitro Release Study

The in vitro release of chloroquine from the freshly prepared niosomes was studied using the dialysis method. Briefly, 1 mL of chloroquine-loaded niosomes was placed in a dialysis bag and immersed in 100 mL dissolution medium (PBS) adjusted to mimic lungs lining pH of ca. 6.9 [19], under stirring at 37 °C. At specified time intervals, 1 mL aliquots were withdrawn and replaced with an equal volume of fresh release medium. Chloroquine concentration was determined spectrophotometrically at *λ*_max_ 343 nm. In vitro release studies were carried out in triplicates, and the results are expressed as means ± SD. To analyze the kinetics of drug release from the niosomal formulation, different kinetic models (zero-order, first-order, Higuchi, and Korsmeyer–Peppas models) were applied, according to Equations (2)–(5) [20]. The model that produced the highest correlation coefficient was used to describe the kinetics of drug release [21].

(2) $$\mathrm{Zero}-\mathrm{order}\;\mathrm{release}\;M_{t}=M_{0}+K_{0}t$$

(3) $$\mathrm{First}-\mathrm{order}\;\mathrm{release}\;\mathrm{Log}\;M=\;\mathrm{Log}\;M_{0}-\frac{K_{1}t}{2.303}$$

(4) $$\mathrm{Higuchi}\;\mathrm{model}\;Mt=kt^{0.5}$$

(5) $$\mathrm{Korsmeyer}–\mathrm{Peppas}\;\mathrm{model}\;\frac{\;M_{t}}{\;M_{\infty }}=kp{\left(t\right)}^{n}$$

in which *M*_0_, *M*_t_, and *M*_∞_ represent chloroquine amounts at time 0, time *t*, and time ∞, respectively. *K*_0_, *K*_1_, *K*, and *K_p_* are the permeation rate constants, and (n) is the permeation rate exponent.

### 2.6. Evaluation of Micromeritic Properties of Lyophilized Drug-Loaded Niosomes/Lactose Blends

Drug-loaded niosomes were prepared and lyophilized with or without the addition of cryoprotectants (e.g., mannitol and trehalose). First, chloroquine-loaded niosomes were prepared, as described previously. The resultant niosomal dispersion was then frozen overnight at −80 °C. Cryoprotectants, mannitol (1% *w/v*) or trehalose (1% *w/v*), were added to the niosomal dispersions prior to freeze-drying. Empty niosomes and chloroquine-loaded niosomes that do not contain cryoprotectants were also used as controls. The samples were then freeze-dried using Alpha 2–4 LD plus freeze-dryer (Martin Christ GmbH, Osterode, Germany). The vacuum pressure was set up at 0.01 mbar and the temperature was maintained at −80 °C for 48 h. Fixed amounts of lyophilized niosomes were then mixed with increasing amounts of a carrier (i.e., lactose) at ratios of 1:1, 1:2, 1:3, and 1:4. The powder blends were evaluated for their flowing properties by measuring the angle of repose, bulk density, tapped density, Carr’s index, and Hausner ratio.

#### 2.6.1. Angle of Repose

The angle of repose of each powder blend was measured using the fixed funnel method. Accurately weighed samples were poured into a funnel, where the height of the funnel was adjusted such that the tip of the funnel just touched the apex of the heap of the powder. The height (h) and diameter (d) of the powder cone were recorded, and the angle of repose was calculated according to the following equation [22]:

(6) $$\tan\;\theta =\frac{h}{r}$$

where h = height of the cone and r = radius of the base.

#### 2.6.2. Bulk and Tapped Density

The powder blend was filled into a 10 mL measuring cylinder. The initial volume was recorded after falling from 2.5 cm height to calculate the bulk density. The cylinder was tapped until powder volume became constant. Bulk and tapped densities were calculated using Equations (7) and (8), respectively [22,23]:

(7) $$\mathrm{Bulk}\;\mathrm{density}=\frac{\mathrm{Weight}\;\mathrm{of}\;\mathrm{powder}}{\mathrm{Bulk}\;\mathrm{volume}}$$

(8) $$\mathrm{Tapped}\;\mathrm{density}=\frac{\mathrm{Weight}\;\mathrm{of}\;\mathrm{powder}}{\mathrm{Tapped}\;\mathrm{volume}\;}$$

#### 2.6.3. Carr’s Compressibility Index and Hausner Ratio

Carr’s index and Hausner ratio were calculated based on the bulk and tapped density, according to Equations (9) and (10) [22,24]:

(9) $$Carr^{\prime }s\;index\;\left(\%\right)=\frac{Tapped\;density-Bulk\;density}{Tapped\;density}\times 100$$

(10) $$Hausner\;ratio=\frac{Tapped\;density}{Bulk\;density}$$

### 2.7. In Vivo Evaluation of the Developed Nanomaterials

The protocol was approved by the Assiut University Animal Ethical Committee (ethical approval number S16-21). Clarithromycin was bought as Klacid^®^ (500 mg clarithromycin lactobionate, Abbott, Cairo, Egypt). Sprague Dawley rats (ca. 200 g) were purchased from Nile Co. for Pharmaceutical and Chemical Industries (Cairo, Egypt). Rats were housed in an air-conditioned atmosphere at 25 °C and maintained on a standardized diet (20% protein, 5% fiber, 3.5% fat, 6.5% ash, and a vitamin mixture) and water ad libitum.

Rats were randomly assigned to three groups (5 rats per group) and treated according to the following protocol: the first group, the control group, was administrated clarithromycin at a dose of 100 mg/kg intravenously 30 min before oral administration of distilled water. Clarithromycin is a CYP3A4 inhibitor used to inhibit the metabolism of chloroquine to allow for accurate detection of chloroquine concentration in blood. The second group was administrated clarithromycin (100 mg/kg) 30 min before oral administration of single-dose chloroquine dissolved in distilled water (62.5 µg/2 mL). The third group was administrated clarithromycin (100 mg/kg) 30 min before intratracheal administration of a single dose of niosomal solution of chloroquine (62.5 µg/0.25 mL). Pulmonary administration was carried out after anesthetizing rats by ketamine at a dose of 5 mg/kg on a horizontal table. The trachea was well illuminated, and intubation was performed by a small plastic cannula. Twenty-four hours following the administration, blood samples were collected from the retro-orbital plexus using capillary tubes on EDTA-coated tubes (Greiner Bio-One, Kremsmünster, Austria), and serum samples were separated via cooling centrifugation at 3000× *g* for 10 min at 4 °C.

#### 2.7.1. Assessment of Chloroquine Concentration in Blood Samples

Chloroquine concentration in blood was assessed via ultra-performance liquid chromatography UPLC-MS/MS (ACQUITY H-Class system, Waters, Milford, MA) using an analytical column (AQUITY UPLC BEH C_18_ 1.7 µm, 2.1 × 50 mm column) at 30 °C, using an injection volume of 5.0 µL and a flow rate of 0.25 mL/min. The mobile phase comprised of 0.2% formic acid in water and acetonitrile at a ratio of 70:30, respectively. This method enabled chloroquine quantification for values as low as 0.2 ng/mL.

A stock standard solution of chloroquine (200 µg/mL) was prepared by dissolving 20 mg of chloroquine in 100 mL (1% formic acid in water–acetonitrile at a ratio of 20:80 *v/v*, respectively). A diluted stock solution at a concentration of 10 µg/mL was then prepared by diluting 5 mL of the initial stock solution in 100 mL of 1% formic acid in water–acetonitrile at a ratio of 20:80 *v/v*, respectively. A calibration curve was constructed using 30 mL of blank rat blood and the previously prepared chloroquine stock standard solution.

The extraction of chloroquine from blood was performed using a standardized experimental procedure. An appropriate number of centrifuge tubes were properly labeled, and 100 µL of the samples (blank, standards, chloroquine, and blood of experimental rats) were dispensed into the centrifuge tubes, followed by the addition of 50 µL of 5% ammonia in water. The samples were vortexed for 30 min using a vortex mixer (Model M37610-33, Thermo Fisher Scientific GmbH, Dreieich, Germany). Then, 1 mL of methyl-tert-butyl ether was added to the centrifuge tubes and vortexed for 3 min. Centrifugation of samples was performed for 20 min at 5000 rpm using a centrifuge (Model 5804, Eppendorf, Hamburg, Germany). A sample of 700 µL from the organic layer was transferred to polypropylene tubes and allowed to evaporate using a vacuum concentrator (Model 5301, Eppendorf) at 45 °C for 30 min. The resultant residue was reconstituted with 200 µL of mobile phase composed of 70% formic acid in water (0.2%) and 30% acetonitrile and vortexed for 30 s before injection of 5.0 µL of the sample to the UPLC-MS/MS. The drug was detected using a tandem mass spectrometer (Model Waters TQD Detector, Waters, Milford, MA, USA) with an ionization source (Model ESI Positive mode, Waters, Milford, MA, USA) and analyzed with Model Masslynxs V4.1 (Waters, Milford, MA, USA).

#### 2.7.2. Assessment of Nephrotoxicity Indices

##### Assessment of Creatinine Level

A commercial kinetic kit was used for the analysis of serum creatinine levels (Biodiagnostic, Cairo, Egypt). In principle, when picric acid is added to creatinine in a sodium hydroxide alkaline solution, a colored complex is formed. Picric acid (20 mmol/L) and sodium hydroxide (1.2 mmol/L) were mixed at equal portions immediately before the assay. Then, 0.5 mL of the mixed working reagents was added to 0.5 mL of a serum sample to form a colored complex that is measured colorimetrically at 520 nm.

##### Assessment of Urea Level

A commercial kit was used for the analysis of urea levels (Biodiagnostic, Cairo, Egypt). Urease was added to the samples and the ammonia produced reacted with a reagent to produce a blue indophenol. The serum sample (0.01 mL) was added to 0.2 mL of phosphate buffer urease (50 mmol/L) and incubated at 37 °C for 5 min. Then, 1 mL of phenol sodium nitroprusside coloring reagent (100 mmol/L) and 1 mL of alkaline reagent sodium hydroxide (150 mmol/L) and sodium hypochlorite (15 mmol/L) were added to the previous mixture and incubated for 10 min at 37 °C. Then, the colored complex was measured colorimetrically at 550 nm against the blank.

#### 2.7.3. Assessment of Hepatotoxicity Indices

##### Assessment of AST Level

A commercial kit was used for the analysis of AST levels (Biodiagnostic, Cairo, Egypt). Serum was added to a mixture of aspartate and α-ketoglutarate, and the formed oxaloacetate was reacted with 2,4-dinitrophenylhydrazine in an alkaline medium. Then, 0.5 mL of glutamic oxaloacetic transaminase buffer substrate was incubated for 5 min at 37 °C, followed by the addition of 0.1 mL of a serum sample to the buffer and incubation at 37 °C for 60 min. Then, 0.5 mL of the coloring reagent 2,4-dinitrophenylhydrazine (1 mmol/L) was added to the mixture and incubated for 20 min at room temperature. Then, 5 mL of sodium hydroxide (0.4 N) was added, followed by incubation for 5 min. The colored complex was measured colorimetrically at 505 nm.

##### Assessment of ALT Level

A commercial kit was used for the analysis of ALT levels (Biodiagnostic, Cairo, Egypt). Serum was added to a mixture of alanine and α-ketoglutarate, and the formed pyruvate was reacted with 2,4-dinitrophenylhydrazine in an alkaline medium. Then, 0.5 mL of glutamic pyruvic transaminase buffer substrate was incubated for 5 min at 37 °C, followed by the addition of 0.1 mL of the serum sample to the buffer and incubation at 37 °C for 30 min. Then, 0.5 mL of the coloring reagent 2,4-dinitrophenylhydrazine (1 mmol/L) was added to the mixture and incubated for 20 min at room temperature. Then, 5 mL of sodium hydroxide (0.4 N) was added, followed by incubation for 5 min. The colored complex was measured colorimetrically at 505 nm.

### 2.8. Statistical Analyses

Values are presented as means ± SDs of at least three independent experiments. Significant differences between groups were evaluated by one-way ANOVA followed by Tukey’s multiple comparison tests (GraphPad Prism 5.0, GraphPad, San Diego, CA, USA). Differences between different groups were considered significant for *p* values less than 0.05.

## 3. Results and Discussion

### 3.1. Preparation and Characterizations of the Niosomal Dispersion

**Figure 1:**
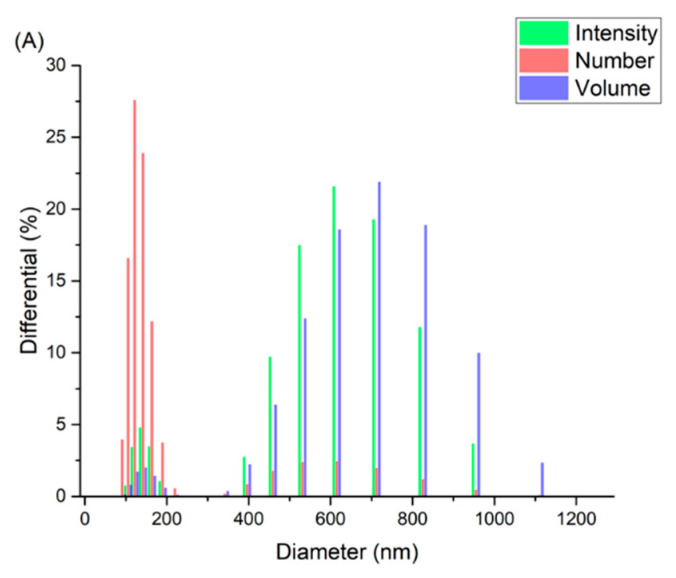

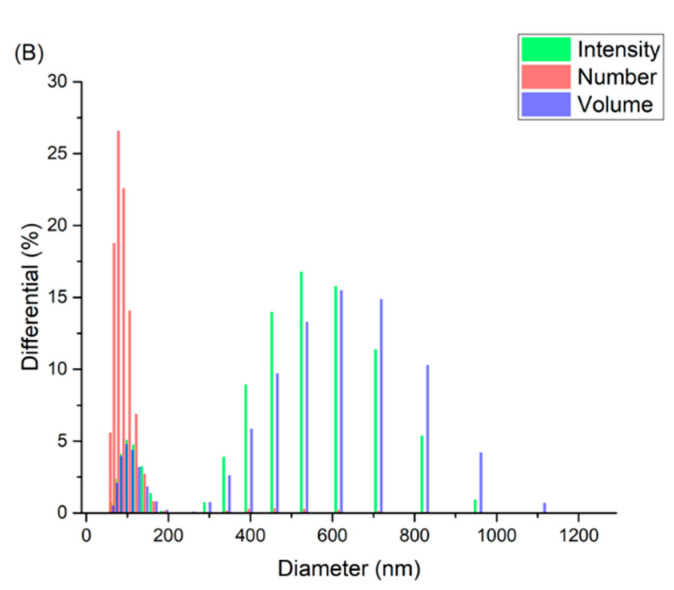
Histograms of the intensity-, volume-, and number-averaged hydrodynamic diameters of (**A**) unloaded and (**B**) chloroquine-loaded niosomal dispersions.

Based on earlier studies performed by our group [7,25] and others [26,27], niosomes composed of cholesterol, Span^®^ 60, and Tween^®^ 40 at a molar ratio of 2:1:1, respectively, were prepared via thin-film hydration method. Nanosized empty and chloroquine-loaded niosomes displayed a mean number-averaged hydrodynamic diameter that ranged from ca. 100–150 nm of PDI ca. 0.6 (Figure 1A,B). However, mean intensity-averaged hydrodynamic diameter showed larger particles (i.e., 608 nm and 506 nm for empty and chloroquine-loaded niosomes, respectively), which might be due to the presence of larger particles in the niosomal dispersion. The size and size distribution characteristics of niosomes are within the acceptable range [28,29]. Zeta potential measures the type and magnitude of electrical charge on the surface of niosomes and is crucial for their physical stability. The presence of charges on the surface of niosomes results in electrostatic repulsion between vesicles and thus imparting greater physical stability [30]. Although surfactants used in this formulation are nonionic themselves (i.e., Span^®^ 60 and Tween^®^ 40), the measured zeta potential values for the empty and drug-loaded niosomes were of relatively high negative values (−44.4 mV and −42.2 mV, respectively), suggesting adequate physical stability of the niosomal dispersion. The negative zeta potential of the particles could be ascribed to the hydroxyl group on the cholesterol molecules [26,31,32]. The calibration curve of chloroquine phosphate solution was established at *λ* = 343 nm, at a concentrations range of 3.5–50 µg/mL. The entrapment efficiency of chloroquine was calculated to be 24.6 ± 0.5%.

**Figure 2:**
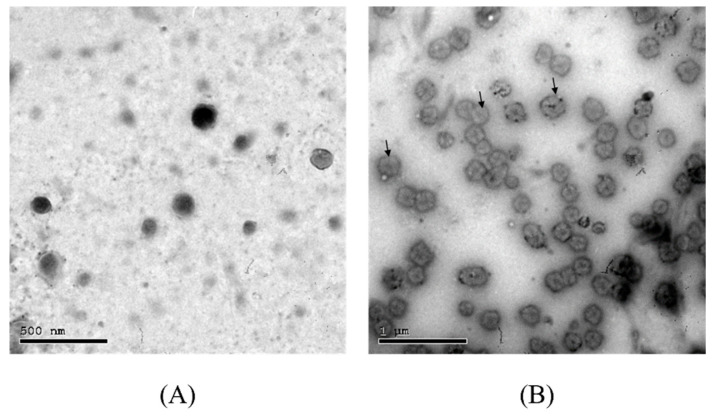
Transmission electron microscopy images of empty (**A**) and chloroquine-loaded niosomes (**B**). Scale bars represent 0.5 and 1 µm, respectively. Particles display rounded shapes with well-defined edges, as demonstrated by the arrows.

The morphologies of empty and chloroquine-loaded niosomes were examined using TEM. The prepared niosomes displayed spherical particles with well-defined edges, as illustrated in Figure 2. The diameters of the niosomal particles (empty and loaded) observed in TEM were in agreement with the size obtained by dynamic light scattering studies.

**Figure 3:**
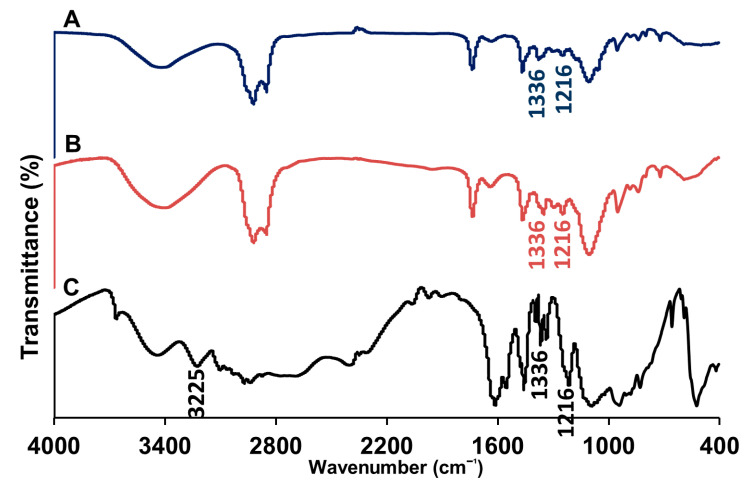
Fourier transform-infrared spectra of chloroquine-loaded niosomes (A), empty niosomes (B), and chloroquine (C).

Fourier transform-infrared (FT-IR) spectroscopic analyses were performed at the range of 4000–400 cm^−1^ to reveal potential chemical and physical interactions between chloroquine and the other ingredients. FT-IR spectra of cholesterol, Span^®^ 60, and Tween^®^ 40 have been explored in earlier studies performed by our group [8]. Figure 3 illustrates the FT-IR spectra of chloroquine, in addition to empty and drug-loaded niosomes. The spectrum of chloroquine showed characteristic peaks at 3225 cm^−1^, which corresponds to N–H stretching, 1336 cm^−1^ and 1216 cm^−1^, which correspond to aromatic C–N stretching and aliphatic C–N stretching, respectively. Empty and chloroquine-loaded niosomes displayed similar spectra, except for additional characteristic peaks of the drug. The spectrum of chloroquine-loaded niosomes exhibited mainly the peaks of niosomes with few overlapping peaks from chloroquine (i.e., 1336 cm^−1^ and 1216 cm^−1^), whereas the N–H stretching peak of chloroquine (i.e., 3225 cm^−1^) disappeared, thus indicating the incorporation of the drug into the niosomes.

**Figure 4:**
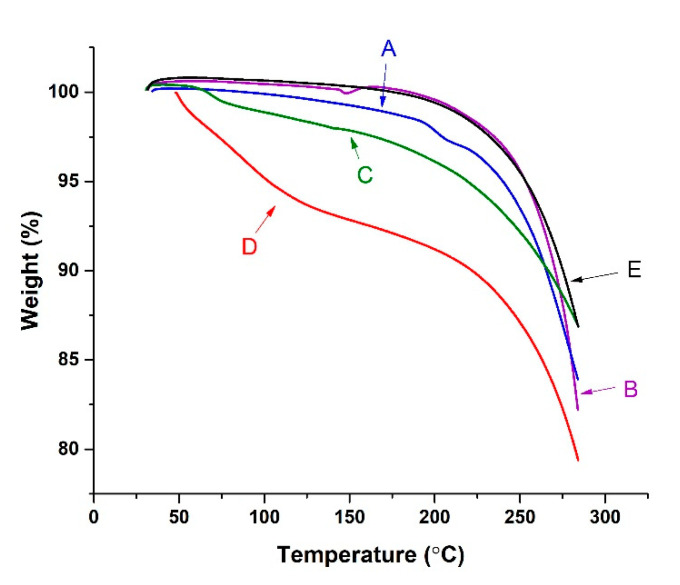
Thermogravimetric analysis (TGA) of chloroquine (A), cholesterol (B), Span^®^ 60 (C), empty niosomes (D), and chloroquine-loaded niosomes (E).

A TGA graph of cholesterol, chloroquine, Span^®^ 60, empty niosomes, and chloroquine-loaded niosomes is presented in Figure 4, which illustrates the initial and final degradation temperatures. The TGA thermogram of chloroquine exhibited small change at 122.0 °C due to water loss from the drug followed by three consecutive weight losses at 192.9 °C (1.3%), 220.2 °C (1.5%), and 283.6 °C (12.9%). The TGA thermogram of cholesterol showed initial degradation at 148.5 °C and reached the maximum at 283.8 °C (17.8% weight loss). The TGA thermogram of Span^®^ 60 showed a degradation at 81.6 °C and a sharp decrease in mass (11.2%) at 284.1 °C. The TGA thermogram of empty niosomes showed that the degradation of empty niosomes started at 126.9 °C and reached its peak at 284.1 °C, and during this step, the empty niosomes lost 14.1% of their total weight. The TGA thermogram of chloroquine-loaded niosomes showed three peaks of weight loss. The first peak is due to loss of moisture at 62.2 °C, the second peak at 160.5 °C is due to thermal degradation (0.6% of its weight), and the third peak at 283.8 °C corresponds to weight loss of 13.2% of the total chloroquine-loaded niosomes weight.

### 3.2. In Vitro Release Studies

**Figure 5:**
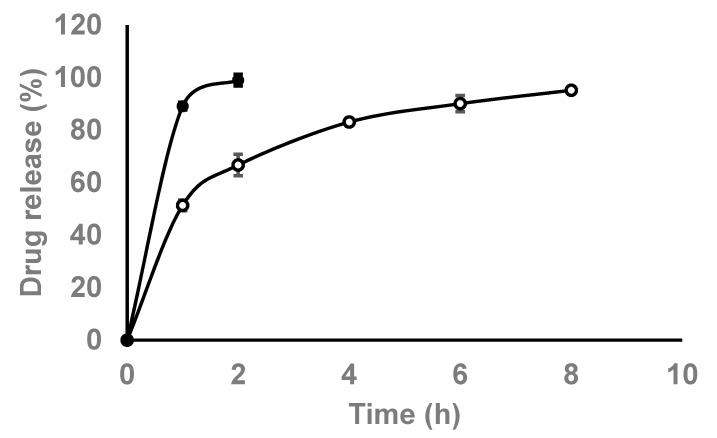
In vitro release of chloroquine, either free (closed circles) or entrapped within niosomes (open circles) over time in PBS at pH 6.9 and temperature of 37 °C.

The drug release study was conducted to determine the mean cumulative drug release percentage of chloroquine, either free or loaded into niosomes, employing the dialysis membrane diffusion technique. Drug release was determined in PBS (pH 6.9 and 37 °C) and the amount of released chloroquine was monitored over 24 h (Figure 5). The percentages of drug release after 2 h were ca. 89 and 51% for the chloroquine-free drug versus the drug loaded into niosomes, respectively. As can be seen in Figure 5, ca. 100% of the chloroquine was released after 2 h in the case of the free drug. However, encapsulation of the drug in niosomes reduced the initial burst release (as compared to the free drug) and provided an extended-release profile up to 8 h.

To investigate the kinetics of drug release from the niosomal dispersion, different kinetic models were applied to the release data. The drug release mechanism was evaluated by curve fitting to various kinetic equations (zero-order, first-order, Higuchi, and Korsmeyer–Peppas). The correlation coefficient (*r*^2^) values for different models are presented in Table 1. It was found that the best fit model for drug release for the chloroquine–niosomal formulation was first-order kinetics.

### 3.3. Evaluation of Powder Flowing Properties (Micromeritic Properties)

Pulmonary drug delivery provides a useful alternative to both oral and invasive parenteral routes of administration for systemic and local effects, particularly for drugs exploited for the management of respiratory diseases. Intratracheal administration and dry powder inhalation are both described in the literature for pulmonary administration of drugs either alone or loaded into different types of nanocarriers. Different methods for the preparation of polymeric and liposomal nanoparticles for administration via dry powder inhalation have been described in the literature, including co-spray drying of the nanoparticles with generally regarded as safe (GRAS) excipients such as lactose and mannitol [33,34]. However, spray drying requires careful considerations, such as optimizing the compositions of excipients and operating conditions for each type of nanocarrier, to allow for the production of particles that exhibit the desired geometric diameters. Alternatively, a simpler approach of carrier-based dry powder inhaler delivery that requires minimal efforts during the formulation processes was reported via blending the inert carrier (e.g., lactose) with the drug-loaded nanoparticles [24,35].

Powder flowing properties, such as the angle of repose, bulk and tapped densities, Carr’s index, and Hausner ratio, are crucial factors that should be carefully considered while designing the optimal dosage form for a designated route of administration. For instance, the manufacturing of tablets requires an extensive evaluation of the flowability of powders or granules to ensure efficient compressibility [36,37]. Assessment of the flowability of lyophilized nanoparticles of different types has been described in the literature for different applications [4,24,38]. To gain an insight into the possibility of inhalational versus intratracheal administration of chloroquine-loaded niosomes, drug-loaded niosomes were lyophilized to obtain dry powder suitable for inhalation. Additionally, the effect of the presence of mannitol and trehalose during lyophilization on the flowability of the resultant powder was also evaluated. Lyophilization of empty and drug-loaded niosomes resulted in the formation of a non-flowable mass. In order to enhance the flowability of lyophilized niosomes, increased proportions of lactose were added to the lyophilized niosomes, mixed, and the flowability of niosomes/lactose powder blends was evaluated.

The flow properties of pharmaceutical powders and blends are crucial characteristics in the preparation of solid dosage forms (e.g., powders). Lactose is a commonly used carrier in dry powder inhalers, owing to its low hygroscopicity, physical and chemical stability, and low cost [39,40]. In this study, chloroquine-loaded niosomes were prepared with the aim of delivering chloroquine via pulmonary administration. To study the possibility of pulmonary administration of the drug-loaded niosomes via dry powder inhalers, the flowability properties of freeze-dried chloroquine-loaded niosomes mixed with lactose as a carrier (at different ratios) were assessed. The effect of incorporation of cryoprotectants, mannitol (1% *w/v*) and trehalose (1% *w/v*), into niosomal dispersions prior to freeze-drying on the flowability of the produced powder blends was also evaluated. The angle of repose, Carr’s index, and Hausner ratio were employed to evaluate the micromeritic properties of the powder blends, and the results are shown in Table 2 [41].

Carr’s index and the Hausner ratio were calculated based on the bulk and tapped densities of the powder blends. Carr’s index measures the tendency of powder to consolidate and has an inverse relationship with flowability (i.e., high compressibility of a material indicates low flowability). A powder with a Carr’s index value < 25 is considered to have good flowing properties. Powders with Carr’s index values ≥ 40 exhibit poor flowability. As shown in Table 2, the freeze-dried niosomes mixed with lactose at different ratios displayed Carr’s index values of 25–40, thus indicating poor flowing properties. Incorporation of higher amounts of lactose was crucial to obtain homogenous powder blends. Although, it could not improve the flowing powder properties. The reduced flowability could be attributed to the reduced particle size upon increasing the amount of lactose. Smaller particles possess large surface areas, which result in enhanced friction and attractive/cohesive forces between particles [39]. This was further supported by the values of the Hausner ratio, as shown in Table 2. The values showed that increasing the amount of lactose resulted in increasing the surface area of the particles and consequently the friction between particles. This in turn worsened the flowability of the respective powders.

The angle of repose was also measured to assess the flowability of the powders. Nevertheless, a different profile was observed. Although increasing the amount of lactose reduced the flowability of the powders, the measured values implied good flowability, in terms of the angle of repose. For instance, freeze-dried chloroquine-loaded niosomes that involved the use of mannitol as a cryoprotectant, when mixed with lactose at a ratio of 1:1, exhibited an angle of repose of 26, which indicates excellent flowability. However, for the same formulation, Carr’s index and Hausner ratio values were 29.1 and 1.4, respectively, which indicated poor flowability. Similar observations were noticed for all formulations when the angle of repose was compared to Carr’s index and the Hausner ratio. The angle of repose, together with other parameters, provides an indication of the flowing properties of the powders and granules. In the case of powders (i.e., small-sized granular materials), the angle of repose is frequently linked with Carr’s index and Hausner ratio values. However, for cohesive materials, measurement of the angle of repose might not reflect the actual flowability of the powders [42].

It is worth noting that the incorporation of different types of cryoprotectants (i.e., mannitol and trehalose) at the same concentration (1%) prior to freeze-drying resulted in the formation of powders of different physical properties, in terms of particle aggregation, stickiness, and flowability. For example, assessment of the flowing properties of the powders (in the presence of mannitol) mixed with lactose was possible at all mixing ratios. On the contrary, assessment of the flowability of powder that involved the use of trehalose prior to freeze-drying was not possible until it was mixed with the carrier (i.e., lactose) at a ratio of 1:3, respectively. This was also the case when chloroquine-loaded niosomes were freeze-dried in the absence of cryoprotectants.

Although the use of a drug-to-carrier ratio of up to 1:67 is justified for the administration of small drug doses (e.g., highly potent drugs) [40], the use of carriers to improve the flowing properties of drug-loaded nanoparticles (already high weights) is usually performed at much lower ratios (e.g., 1:1.5) [43]. Furthermore, the formed particles could not be easily dispersed via aerosolization, because they existed mainly as agglomerates, probably due to the lipid content of niosomes that increased the cohesiveness of the lyophilized mass. Administration of the formulation using a dry powder inhaler was excluded due to the poor flowability of the freeze-dried niosomes mixed with lactose, diversity in the results, and the need to include high amounts of the carrier to obtain homogenous powder distribution. Alternatively, intratracheal administration was examined.

### 3.4. In Vivo Valuation of the Chloroquine-Loaded Niosomes

The potential use of chloroquine is supported by its low cost, availability, and acceptable safety and tolerability within the recommended doses [44]. However, chloroquine and its derivatives possess a high volume of distribution upon oral administration (i.e., 100 L/kg). This indicates a high tissue distribution and therefore requires oral administration of relatively high doses, which leads to the associated side effects (e.g., retinotoxicity, neurotoxicity, myotoxicity, etc.) [16]. Cardiotoxicity is the main concern with prolonged use, especially in patients with hepatic or renal dysfunction [45,46]. This may in turn hamper the successful reuse of chloroquine for other clinical applications (e.g., COVID-19). Hence, localized administration of drugs via intratracheal administration provides a promising strategy towards improving efficacy while reducing systemic side effects.

**Figure 6:**
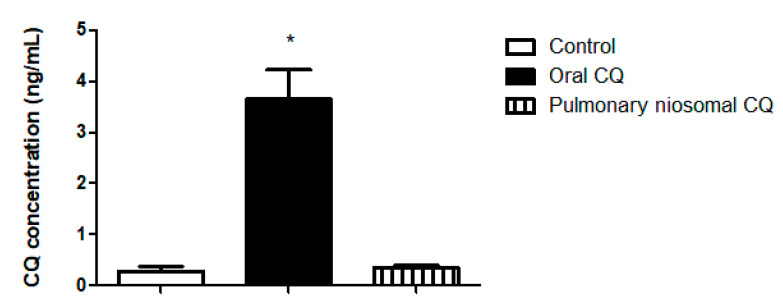
Chloroquine (CQ) concentrations in the blood of the treated animals following oral and intratracheal administration of drug solution and drug-loaded niosomes, respectively. Values are illustrated as means ± SDs (*n* = 5). The * denotes statistical significance from the control group (*p* < 0.0001) using a one-way analysis of variance (ANOVA), followed by Tukey–Kramer as a post-hoc test.

**Figure 7:**
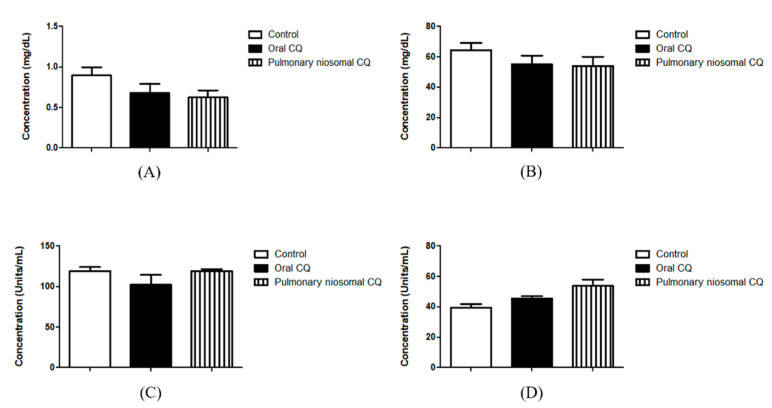
Creatinine (**A**), urea (**B**), AST (**C**), and ALT (**D**) levels in blood of the treated animals following oral and intratracheal administration of chloroquine (CQ) drug solution and drug-loaded niosomes, respectively.

As a proof of concept, a preliminary investigation was performed to study the efficiency of intratracheal administration of chloroquine-loaded niosomes to enhance local drug delivery into the lungs while reducing systemic exposure. As nanoparticles have already been found to prolong the residency of loaded drugs into the lungs [6], preliminary investigation was limited to the administration of drug-loaded nanoparticles to reduce the number of animals in use. Chloroquine concentration in blood was assessed via ultra-performance liquid chromatography UPLC-MS/MS using an analytical column at 30 °C. The equation of the calibration curve in this study was y = 152.843 x + 8.76694 (correlation coefficient = 0.9977). The linearity of the calibration curve was from 0.2 to 100 ng/mL. The lower limit of detection was 0.2 ng/mL, and the accuracy was 100.05 ± 6.264%. Upon comparing intratracheal and oral administration of chloroquine-loaded niosomes and chloroquine, respectively, chloroquine concentration was significantly higher (approximately 10 folds) in blood when administrated orally compared to administration of the niosomal dispersion through the trachea (Figure 6). The reduced systemic exposure to the drug observed in our study could be due to a combined effect between localized delivery of the drug into the lungs and sustained release from the nanoparticles. The larger size of particles compared to the drug’s small molecules aids in prolonging the residence of the drug in the lungs [6]. Hence, the developed formulations might have a great potential for pulmonary administration of chloroquine and other drugs to maximize local drug concentration in the lungs while reducing systemic exposure. Applications might be also extended to other antiviral and antibacterial drugs used for the treatment of respiratory tract infections. There were no statistical differences between groups when nephrotoxicity (creatinine and urea levels) and hepatotoxicity (AST and ALT) indices were assessed (Figure 7), thus providing initial evidence of biocompatibility of the developed therapeutic system.

## 4. Conclusions

In the current study, chloroquine-loaded niosomes were successfully prepared and characterized. The developed niosomes demonstrated a nanosized spherical morphology and displayed an entrapment efficiency of ca. 24.5%. To examine the possibility of aerosolization of the drug-loaded niosomes, a lyophilized product was prepared. However, they exhibited poor flowability that was not sufficiently improved after the addition of lactose or when cryoprotectants were used throughout the lyophilization process. Consequently, intratracheal administration was exploited for pulmonary administration of the chloroquine-loaded niosomes, rather than the use of dry powder inhalers. To examine the ability of developed nanocarriers to reduce systemic drug exposure, in vivo studies were carried out and revealed a 10-fold lower concentration in blood following intratracheal administration of the drug-loaded niosomes compared to oral administration of the free drug. Hence, the use of a dry powder inhaler for lyophilized drug-loaded niosomes is not recommended, whereas intratracheal administration might provide a promising strategy for pulmonary administration of niosomal dispersions while minimizing systemic drug exposure and adverse reactions.

## Figures and Tables

**Table 1:** Kinetic models of chloroquine release from niosomes.

Kinetic Model	Correlation Coefficient (*r*^2^)
Zero-order	0.7302
First-order	0.9814
Higuchi	0.9395
Korsmeyer–Peppas	0.9564

**Table 2:** Micromeritic properties of lyophilized empty niosomes and chloroquine-loaded niosomes mixed at various weight ratios with lactose (1:1–1:4), in terms of angle of repose, Carr’s index, and Hausner ratio. The effect of the presence of cryoprotectants, namely mannitol and trehalose, during the lyophilization process on the micromeritic properties was also studied. Values are presented as means ± SDs.

Formulation	Niosomes: Lactose Ratio	Angle of Repose	Carr’s Index	Hausner Ratio
Empty Niosomes	1:1	N/A	N/A	N/A
	1:2	N/A	27.5 ± 1.2	1.4 ± 0.0
	1:3	26.0 ± 0.3	25.4 ± 0.5	1.3 ± 0.0
	1:4	31.8 ± 1.2	31.6 ± 0.6	1.5 ± 0.0
Chloroquine-loaded Niosomes	1:1	N/A	N/A	N/A
	1:2	N/A	N/A	N/A
	1:3	N/A	36.8 ± 0.0	1.6 ± 0.0
	1:4	28.7 ± 0.3	36.5 ± 0.3	1.6 ± 0.0
Chloroquine-loaded Niosomes (1% mannitol)	1:1	26.0 ± 1.1	29.1 ± 1.0	1.4 ± 0.0
	1:2	33.68 ± 0.3	27.9 ± 3.1	1.4 ± 0.1
	1:3	39.2 ± 0.9	32.9 ± 2.0	1.5 ± 0.1
	1:4	39.7 ± 4.1	40.3 ± 0.7	1.7 ± 0.0
Chloroquine-loaded Niosomes (1% trehalose)	1:1	N/A	N/A	N/A
	1:2	N/A	N/A	N/A
	1:3	33.7 ± 0.5	33.3 ± 1.4	1.5 ± 0.0
	1:4	34.3 ± 0.8	38.2 ± 3.4	1.6 ± 0.1

## Data Availability

Not applicable.
